# Introducing the Needs in Recovery Assessment (NiRA) into clinical practice: protocol for a pilot study investigating the formal and systematic assessment of clinical and social needs experienced by service users at a tertiary, metropolitan mental health service

**DOI:** 10.1186/s40814-021-00919-8

**Published:** 2021-09-30

**Authors:** Ellen L. Davies, Andrea L. Gordon, Kenneth J. Hooper, Robert E. Laing, Elizabeth A. Lynch, Lemuel J. Pelentsov, Adrian J. Esterman, Gillian Harvey

**Affiliations:** 1 Adelaide Nursing School, The University of Adelaide, Level 4 AHMS Building, 5 North Terrace, Adelaide, South Australia 5001 Australia; 2 School of Biomedicine, The University of Adelaide, Level 3 Helen Mayo South, Frome Street, Adelaide, South Australia 5001 Australia; 3 Youth Mental Health Service, SALHN, GP Plus Marion, 10 Milham St., Oaklands Part, South Australia 5046 Australia; 4 Caring Futures Institute, Flinders University, Sturt Rd, Bedford Park, South Australia 5042 Australia; 5 Clinical and Health Services, University of South Australia, Centenary Building, North Terrace, Adelaide, South Australia 5001 Australia

**Keywords:** Pilot study, Needs assessment tool, Mental health, Recovery, Protocol

## Abstract

**Background:**

The Needs in Recovery Assessment (NiRA) is a newly developed needs assessment tool, designed to identify the needs of people recovering from mental illness. This tool has been evaluated outside of the clinical context for validity and reliability. The aim of this study is to introduce the NiRA into clinical practice and to evaluate the value of the NiRA as an adjunct to service delivery from the perspectives of stakeholders and to evaluate the barriers and facilitators of embedding the NiRA in a mental health service.

**Methods:**

The establishment of the NiRA in a tertiary mental health unit over a 6-month period will be evaluated using a multi-methods approach. Quantitative data will be collected using the NiRA itself and the Recovery Self-Assessment (RSA). Face-to-face interviews with service users and clinicians will be conducted following the initial completion of the NiRA, with a follow-up interview for service users on discharge from the service. Regular informal follow-up with clinicians throughout the study will support the introduction of the NiRA. Descriptive statistics will be used to analyse quantitative data, and descriptive qualitative methods will be used to analyse data from interviews.

**Discussion:**

Aligning mental health services with recovery-oriented frameworks of care is imperative. The NiRA is a tool that has been designed in accordance with recovery principles and may assist services to be more recovery-oriented. If the NiRA is able to achieve the aims and objectives of this project, a larger implementation study will be conducted.

Trial registration

Australian and New Zealand Clinical Trial Registry (ANZCTR), ACTRN12621000316808

**Supplementary Information:**

The online version contains supplementary material available at 10.1186/s40814-021-00919-8.

## Introduction

### Background

Translation of recovery-oriented principles into tertiary mental health services in Australia, and internationally, is an ongoing process that has been hindered by a number of internal and external factors [1]. Arguably, one of the barriers to the implementation of recovery-oriented care delivery is the assessment tools that are used in mental health services, which have received criticism for their lack of recovery and person-centred focus [2, 3].

In Australia, recovery-oriented frameworks and mental health policy clearly identify the requirement of mental health services to assess and address the clinical and social needs of people who present to mental health services [4, 5]. Despite this requirement, the consistent use of a needs assessment tool in mental health services to formally and systematically document needs, as well as approaches taken to address needs, has not been recorded in academic literature. On the contrary, anecdotal evidence suggests that needs are assessed in an ad hoc manner [6].

Recognising the known limitations of the current assessment tools, a mixed methods research project was undertaken in 2019 to develop, validate and test the reliability of a new recovery-oriented assessment tool. This tool is designed to facilitate the identification and prioritisation of needs experienced by individuals recovering from an episode of mental illness [7].

The Needs in Recovery Assessment (NiRA) comprises 38 items of need, including a range of practical, daily activity, physical, informational, emotional, psychological and relationship needs. Its development has been informed by previous research that has investigated the needs of individuals recovering from the first episode of mental illness and by service users and clinicians who participated in focus groups and workshops to develop and design the tool [6, 8] (see Additional File 1).

The NiRA includes three sections that facilitate discussions between mental health service users and mental health clinicians [9]. Section 1 evaluates the relevance of the 38 items of need to the participating service user. Each item of need is rated on a 5-point scale, which in turn prompts a conversation about prioritising identified unmet needs. Section 2 facilitates a discussion regarding the prioritisation of identified needs. Section 3 guides a follow-up discussion on how to meet these needs. This can be arranged for a mutually agreed time (e.g. 1 or 2 weeks), and the plan to address needs can be revised or reoriented if required.

**Fig. 1:**
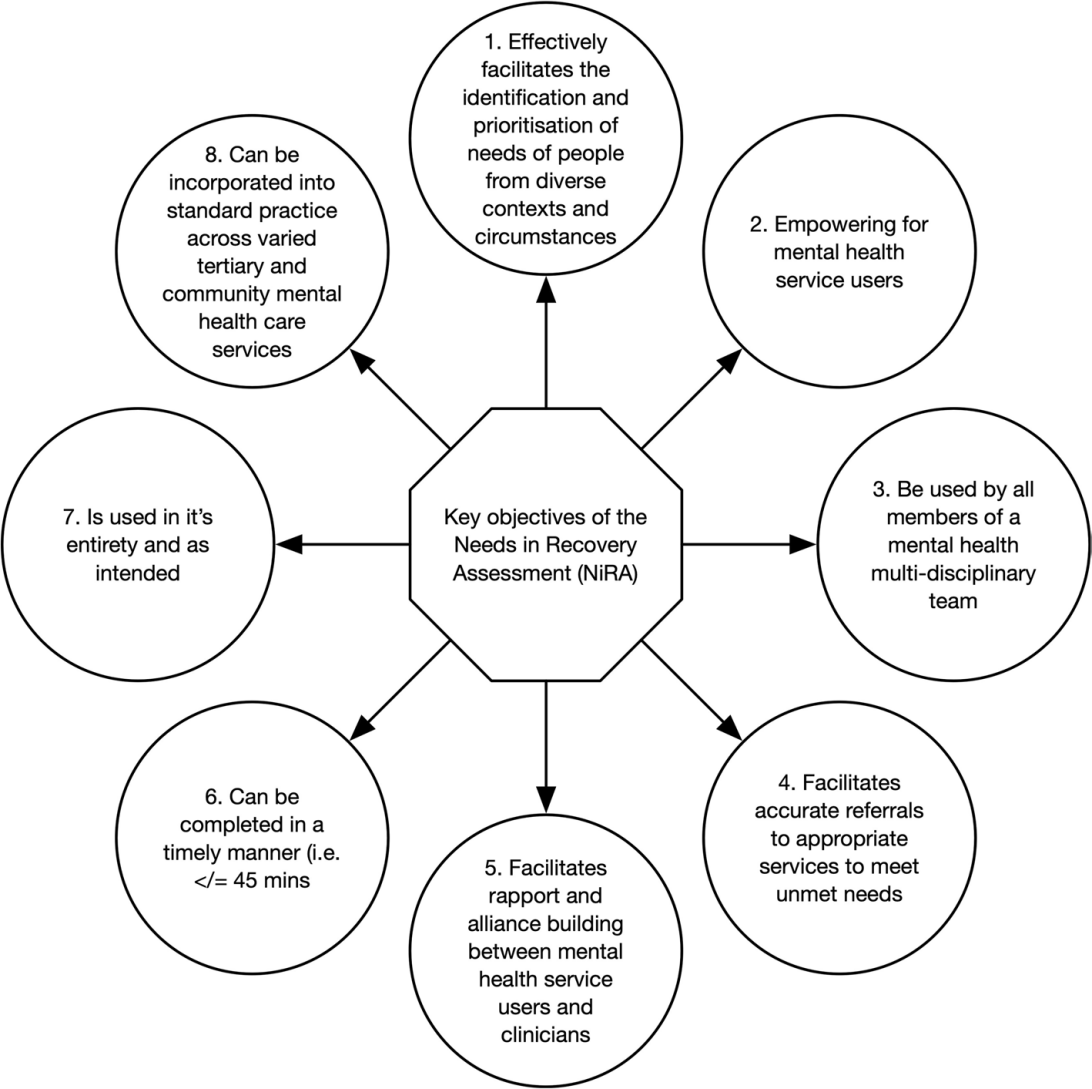
Key objectives of the Needs in Recovery Assessment (NiRA)

A number of guiding principles were considered during the NiRA design process, resulting in the eight key objectives in Fig. 1 [7]. To date, the NiRA has not been trialled in a clinical setting. This pilot study will evaluate the capacity of the NiRA to meet these key objectives when it is introduced into clinical practice.

Project aims

The aims of this project are to evaluate the value of the NiRA to enhance recovery-oriented service delivery and to evaluate the barriers and facilitators of embedding the NiRA in a mental health service. The primary research questions for this study are as follows:

1. To what extent can the NiRA assist staff to provide recovery-oriented mental health services?
2. What are the barriers and facilitators for introducing the NiRA into clinical practice?

Secondary research questions will elicit more specific information required to adequately address the following primary research questions:

I. Is the NiRA effective in identifying and prioritising the needs of individuals recovering from an episode of mental illness?
II. Does the NiRA facilitate an increased sense of empowerment in the planning and implementation of person-centred care and recovery for mental health service users?
III. Does the NiRA enable services to better align with recovery-oriented frameworks of care?
IV. Does the NiRA facilitate effective and appropriate referrals to other services?
V. Is the NiRA effective in enhancing rapport and alliance building between service users and clinicians?
VI. When is the optimal point in the service user’s journey to introduce the NiRA?
VII. What differences exist in the experience of using the paper-based NiRA and the electronic version of the NiRA (eNiRA)

## Methods

The proposed study is a multi-methods project, designed in accordance with recommendations for reporting protocols of pilot and feasibility trials by Thebane and Lancaster [10].

### Trial design

The proposed pilot study will evaluate the use of the NiRA in a clinical setting and identify the potential implementation challenges [11].

### Participants

Service users and clinicians will be recruited from one metropolitan tertiary mental health unit. Service users include individuals who have been admitted to the participating service. Mental health clinicians from the multi-disciplinary team employed by the unit may include registered nurses, occupational therapists, social workers, psychologists and psychiatrists. The inclusion and exclusion criteria for service users and clinicians have been outlined in Table 1.

**Table 1:** Participant selection criteria

Inclusion criteria	Exclusion criteria
*Service users*	
1. Service user of the participating mental health service at the time of recruitment 2. > 16 years of age 3. Considered by a senior member of the mental health team to be recovering from an episode of mental illness^a^ 4. Considered by a senior member of the mental health team to have the capacity to consent to participate in the study^a^ 5. Parent/guardian signed consent if < 18 years of age	1. Currently under a community treatment order, forensic orders or Department of Child Protection intervention order 2. Diagnosis of intellectual disability or history of a significant brain injury
*Clinicians*	
1. Any clinical member of the mental health team in the participating service 2. Signed consent form	1. Clinicians who are members of the research team

^a^The purpose of these inclusion criteria is to ensure that participants are not experiencing symptoms of a mental illness that will likely impair decision-making capacity or ability to provide informed consent. Clinicians will use their clinical experience and expertise to assess the individual’s mental state and use their clinical judgement to decide whether it is appropriate to invite the participant into the study

### Interventions

The original paper-based NiRA, as well as a multi-device compatible electronic version of the tool (the eNiRA), will be administered in a tertiary metropolitan mental health service in Adelaide, Australia. Data collection will continue over a 6-month period. Participants will engage with the NiRA throughout this period.

### Data collection tools

This is a multi-methods study where a combination of qualitative and quantitative data will be collected. The demographic information that will be collected from participants is included in Table 2.

**Table 2:** Demographic information to be collected

Service users	Clinicians
Age (range)	Age (range)
Gender (self-identified)	Gender (self-identified)
Length of admission to service (from service records)	Profession
	Duration working in mental health services

### Quantitative data

The Recovery Self-Assessment (RSA) is a validated set of questionnaires designed to evaluate the degree to which recovery-oriented practices are implemented in a service from multiple perspectives, including service user and clinician perspectives [12]. Each questionnaire contains 36 items that are rated on a 5-point Likert scale. Items are divided into five categories, including *life goals*, *involvement, diversity of treatment options*, c*hoice* and *individually tailored services* [12]. The RSA has been identified as an appropriate tool for evaluating recovery-oriented practices in the Australian mental health context [13, 14].

Prior to the introduction of the NiRA, service users and clinicians will complete the RSA-Person in Recovery and RSA-Provider questionnaires, respectively. The purpose of administering these questionnaires is to gauge the degree to which participants perceive the service to be recovery-oriented. These questionnaires will be re-administered towards the end of the study. Whilst there are likely to be too many variables to directly attribute changes in responses on the RSA questionnaires to the introduction of the NiRA, the questionnaire will provide valuable data regarding the perceptions of service users and clinicians towards recovery-oriented practices within the service. Extreme caution will be exercised in drawing absolute conclusions about the effectiveness of the NiRA to change this perception. The use of the RSA will simply be used to understand how service delivery is perceived over the duration of the study. An electronic version of the RSA questionnaires will be provided through SurveyMonkey®. Written permission to use the RSA has been provided by the authors.

Data regarding the type and urgency of need, priorities for meeting needs, plans to address needs, referrals to other services and the follow-up discussion points will be collected from all NiRA forms administered throughout the study period. These data will be de-identified at the time of collection, with the following information retained: gender, age, date of assessment and a de-identified list of people who attended the session (for example, service user, mental health clinician, mother, father).

### Qualitative data

Face-to-face interviews will be used to collect qualitative data. Service users will be invited to participate in between one and three face-to-face interviews structured to document experiences of needs, and to map individuals’ journeys through the health system. Patient journey mapping tools designed by Kelly et al. [15] will be modified to guide interviews and to document service users’ journey and experience of the mental health service. Examples of questions that will be asked in these interviews are included in Table 3.

**Table 3:** Guiding questions for face-to-face interviews with clinicians during the trial of NiRA

	Clinicians		Service users
1	Can you describe your past and current experiences of assessing service users’ needs?	1	What were your experiences of entering the mental health service?
2	What challenges did you experience when using the NiRA?	2	What has your experience of being admitted into this mental health service been like?
3	How do you think the service users felt about being asked about their needs in this way?	3	What, if any, discussions have you had about any needs that you are experiencing with mental health clinicians?
4	What enabled the NiRA to be introduced into this unit?	4	What has helped your experience of recovering from a mental illness?
5	What challenges do you think the unit had when the NiRA was introduced?	5	What has prevented you from receiving the assistance that you have felt you needed to recovery?
6	What barriers do you believe may impede the introduction of the NiRA into other mental health services?	6	What is the impact of having conversations about your specific needs?

The first interview will take place 2 to 4 weeks after the initial NiRA assessment has been undertaken to identify needs with the participant. The second and third interviews will be arranged at monthly intervals. Service users will be invited to review analysed and synthesised data for accuracy.

Patient journey mapping will be conducted, to provide a rich description of the experiences of service users as they enter into the service. The experience of met and unmet needs will be identified and discussed, with data obtained from interviews compared with data from the NiRA forms completed with clinicians. This will highlight the extent to which the NiRA is being used to identify service users’ needs and the accuracy with which it is being completed. The interviews will also provide insight into the service user experience of being asked about their needs formally and systematically.

Interviews with mental health clinicians will also be undertaken. The purpose of these interviews is to explore, from the clinicians’ perspective, the value of the NiRA in understanding service user needs and to explore this in the context of service users’ journeys through the health care system. These semi-structured interviews will be guided by the questions listed in Table 3 and conducted in the fourth and fifth months of the trial and contribute to evaluating the feasibility of introducing the NiRA into other mental health care services.

Implementation of the NiRA into practice will be an iterative and negotiated process. The members of the research team will meet regularly with clinicians to discuss the most appropriate way to introduce the tool and to support group decision-making. Regular, informal follow-up with clinicians will also continue throughout the trial to support the introduction of the tool and to address concerns and questions that clinicians may have. Researcher notes will be recorded electronically throughout the trial period and used during data analysis to describe the barriers and facilitators to the implementation of the NiRA into clinical practice.

### Outcomes

The outcomes that will be measured relate to the aims of this study and are included in Table 4.

**Table 4:** Measurable outcomes of the pilot study

Primary outcome	Data collection	Data analysis	Associated research question
**Primary outcome**			
1. Type, volume, severity and prioritisation of needs that are reported by service users	Completed NiRA forms	Descriptive statistical analysis	PQ 1 SQ I
	Interviews with service users	Descriptive qualitative analysis	
**Secondary outcomes**			
1. Perceived barriers and facilitators for introducing the NiRA into clinical practice	Interviews with service users and clinicians Researcher observation notes	Descriptive qualitative analysis	PQ 2 SQ III, VII
2. Perceptions of mental health service users and clinicians regarding the service’s capacity to deliver recovery-oriented care prior to and after the introduction of the NiRA	Recovery Self-Assessment questionnaires	Descriptive statistical analysis	PQ 1 SQ I, II, III, V
	Interviews with service users and clinicians	Descriptive qualitative analysis	
3. Service users’ perception of empowerment and satisfaction relating to how needs are assessed	Interviews with service users and clinicians	Descriptive qualitative analysis	PQ 1 SQ I, II, V, VI
4. Appropriateness of referrals made by clinicians for service users when needs are identified	NiRA forms Interviews with service users—patient journey mapping discussions	Compare needs with referral type Descriptive qualitative analysis	PQ 1

*PRQ* primary research question, *SRQ* secondary research question

The feasibility of introducing the NiRA into other clinical settings will be explored and described in the eight domains identified by Bowen et al. [16], comprehensive of acceptability, demand, implementation, practicality, adaptation, integration, expansion and limited-efficacy testing. Data that has been gathered from all data collection points (quantitative and qualitative) will be collated under these domains. Barriers and facilitators that are identified by service users and mental health clinicians will be described in the context of these domains, with limitations of statistical power acknowledged.

### Sample size

A minimum of ten service users and 80% of clinicians employed by the participating service (*n* = 9) will be recruited to complete the pre- and post-trial questionnaires. Ideally, the same service users will also complete the post-trial questionnaires, but this may not be possible as a result of the admission time frame on the unit. We aim to have 80% of all service users who attend the service assessed with the NiRA over the 6-month trial period. The number of service users attending the service varies, but we estimate that between 40 and 80 service users will participate in at least one assessment with the NiRA. We aim to map the journeys of a minimum of five service users over the 6-month trial period.

### Analytical methods

Descriptive statistics, including counts, means and percentages, will be used to analyse the responses from questionnaires and from the collated NiRA forms. Analysis of RSA questionnaires will be weighted in accordance with the author’s recommendations [12].

Qualitative descriptive analysis of data from face-to-face interviews with service users and clinicians will be undertaken using the methodological approaches and principles outlined by Milne and Orbele [17] and Sandelowski [18]. Member checking for patient journey mapping interviews will be offered to service users. They will have the opportunity to review, alter and comment on transcripts. Verbatim transcripts from all interviews with clinicians and service users will be viewed by a minimum of two members of the research team. A minimum of two transcripts from each of these groups will be coded by two researchers to pilot codes. One researcher will be responsible for coding the remainder of the transcripts. All members of the research team will participate in analysing codes and developing and reviewing themes and sub-themes.

Qualitative and quantitative data will be drawn together in the final analysis, with observations from all methodologies collated and presented in manuscripts for publication.

### Participant flow

Anticipated participant flow through this study is described in Table 5, as per the SPIRIT guidelines [19, 20].

**Table 5:** Participant flow

Time point	Enrolment	Post-allocation			Trial end
	− *t*_1_	*t*_1_	*t*_2_	*t*_3_	*t*_4_
**Enrolment**					
Eligibility screen	X		X	X	
Informed consent	X		X	X	
**Interventions**					
Education (clinicians)		X			
NiRA included in clinical practice			X	X	
**Assessments**					
Baseline data collection	X	X			
Pre-trial questionnaire (service users)		X			
Pre-trial questionnaire (clinicians)		X			
Needs assessment with service users with NiRA			X	X	
Journey mapping (service users)		X	X	X	
Face-to-face interviews (clinicians)			X	X	
Post-trial questionnaire (service users)					X
Post-trial questionnaire (clinicians)					X

*− t*_*1*_ recruitment into study, *t*_*1*_ baseline data collection, *t*_*2*_ trial of NiRA in clinical setting, *t*_*3*_ peri-trial data collection (6 months), *t*_*4*_ post-trial data collection

### Recruitment

Participants will be recruited from one tertiary, metropolitan mental health unit. The unit is relatively small compared to others in the region and provides outpatient services, group therapy and case management services. The unit comprises a multi-disciplinary team of clinicians, comprehensive psychiatrists, clinical psychologists, mental health nurses, social workers and occupational therapists.

### Service users

Prior to introducing the NiRA into practice for the trial period, all service users who meet the inclusion criteria will be asked by a member of the mental health team if they are interested in completing the RSA questionnaires. Contact details of service users who consent to complete the RSA will be provided to the research team who will contact the prospective participant to discuss participation and the consent process.

Service users will be purposively recruited to participate in the face-to-face interviews. A member of the mental health team will approach service users who meet the inclusion criteria to determine their interest in participating. Contact details of any service users who consent to participate in journey mapping will be provided to the research team who will contact the prospective participant to discuss participation in journey mapping and the consent process.

For any service users who are aged between 16 and 18, signed consent from both the service user and their parent or guardian will be a pre-requisite for completing demographic and RSA questionnaires. Prospective participants in this age bracket will be asked to provide the contact details of a parent or guardian who will be informed of the study requirements, process and time commitment. Only service users aged > 18 will be eligible to participate in the face-to-face interviews.

All mental health service user participants will receive a $10 electronic voucher for completing demographic and RSA questionnaires and a $50 electronic voucher for each face-to-face journey mapping interview that will last a maximum of 75 min.

### Clinicians

All clinicians employed at the unit where the NiRA will be introduced will be invited to complete the questionnaires prior to and after the trial period (see Table 4) via an email sent by the lead researcher (ED). A participant information form will be included in this email, and informed consent will be implied if a questionnaire is completed. A reminder email will be sent 2 weeks after the initial email.

All clinicians who work for the unit will be invited to participate in face-to-face interviews with a member of the research team who is not connected with the service in any other way. Signed consent will be a pre-requisite for participating in face-to-face interviews.

### Harms

Any serious adverse events that are reported by participants as a result of completing questionnaires, or participating in face-to-face interviews, will be reported to the ethics committee within 7 days of the incident being known.

## Discussion

The purpose of this pilot study is to evaluate the value of the NiRA when it is used as an adjunct to care in a tertiary mental health unit. Alongside data from service users and clinicians that relates to the tool itself, data relating to implementation challenges will be collected and analysed to evaluate and describe the barriers and facilitators that may affect the introduction of the NiRA to other mental health units and services.

Limitations of the study will include the relatively small sample sizes that will be recruited from a single site public metropolitan tertiary mental health service over the 6-month trial period. The results of the trial will not be generalisable to the broader Australian or global population.

## Supplementary Information

**Additional file 1.** The Needs in Recovery Assessment (NiRA) has been developed to facilitate a conversation about unmet needs you may be experiencing.

## Data Availability

Not applicable

## Abbreviations

eNiRA: Electronic Needs in Recovery Assessment
NiRA: Needs in Recovery Assessment
RSA: Recovery Self-Assessment
SPIRIT: Standard Protocol Items: Recommendations for Interventional Trials
