# Supplementary material for: Introducing the Needs in Recovery Assessment (NiRA) into clinical practice: protocol for a pilot study investigating the formal and systematic assessment of clinical and social needs experienced by service users at a tertiary, metropolitan mental health service
Source: Pilot Feasibility Stud. 2021 Sep 30;7:181. doi: 10.1186/s40814-021-00919-8 (PMC8482663; doi:10.1186/s40814-021-00919-8)
Supplement: Supplementary file 1 — Additional file 1. The Needs in Recovery Assessment (NiRA) has been developed to facilitate a conversation about unmet needs you may be experiencing. [file 40814_2021_919_MOESM1_ESM.pdf]

Needs in Recovery Assessment

Date of assessment:
Place of assessment:
Service user name:
Service user ID/URN:
Lead mental health clinician:
Others present:

Introduction for service users

The Needs in Recovery Assessment (NiRA) has been developed to facilitate a conversation about unmet needs you may be experiencing. There are three sections in this assessment:

- Section 1 provides an opportunity for unmet needs to be identified and rated. Some of these needs may apply to you, but many will not be relevant.
- Section 2 provides an opportunity for unmet needs to be prioritised and for us to discuss a plan for meeting those needs. You may have the resources and support to meet these needs, but if you don't and would like some assistance, we can discuss options.
- Section 3 will be completed in a follow-up appointment so we can discuss progress with meeting needs. This should happen in the next 1 to 2 weeks.

We want to support you in exploring and understanding your needs, but we also understand that sometimes needs can be difficult to discuss. If you don't want to discuss particular needs today, there is the option of ticking the 'revisit later' box in Section 1 and we can talk about it when you are ready.

Section 1 – Identifying unmet needs

1 = No need; 2 = Some need; 3 = Moderate need; 4 = Significant need; 5 = Urgent need; NA = Not applicable

Practical needs

How would you rate your need for support with the following?	1	2	3	4	5	NA	Revisit later
Safe accommodation	☐	☐	☐	☐	☐		☐
Stable accommodation	☐	☐	☐	☐	☐		☐
Method to contact people (e.g. phone, internet)	☐	☐	☐	☐	☐		☐
Sufficient income	☐	☐	☐	☐	☐	☐	☐
Support with social services (e.g. housing trust, welfare services, child support, legal aid)	☐	☐	☐	☐	☐	☐	☐

Comments

Daily activity needs

How would you rate your need for support with the following tasks?	1	2	3	4	5	NA	Revisit later
Preparing or getting meals	☐	☐	☐	☐	☐		☐
Shopping for household goods (e.g. food, clothing)	☐	☐	☐	☐	☐	☐	☐
Cleaning and maintenance of your home	☐	☐	☐	☐	☐	☐	☐
Managing income	☐	☐	☐	☐	☐	☐	☐
Self-care (e.g. hygiene, appearance)	☐	☐	☐	☐	☐		☐
Managing medication routine	☐	☐	☐	☐	☐	☐	☐
Transportation (e.g. to & from school, work, appointments)	☐	☐	☐	☐	☐		☐
Attending place of work or study	☐	☐	☐	☐	☐	☐	☐
Performing well at work or place of study	☐	☐	☐	☐	☐	☐	☐
Knowing what to do during the day (e.g. having a routine, creative outlet, education or employment)	☐	☐	☐	☐	☐		☐
Care of dependent(s) (e.g. children, other relatives, pets)	☐	☐	☐	☐	☐	☐	☐

Comments

1 = No need; 2 = Some need; 3 = Moderate need; 4 = Significant need; 5 = Urgent need; NA = Not applicable

Physical health needs

How would you rate your need for support with the following?	1	2	3	4	5	NA	Revisit later
Managing physical health and/or illness	☐	☐	☐	☐	☐		☐
Managing side-effects of medications	☐	☐	☐	☐	☐	☐	☐
Maintaining a healthy body weight	☐	☐	☐	☐	☐		☐
Maintaining healthy eating habits	☐	☐	☐	☐	☐		☐
Maintaining healthy sleeping patterns	☐	☐	☐	☐	☐		☐
Attending regular GP appointments (e.g. review of physical & sexual health, medication reviews)	☐	☐	☐	☐	☐		☐
Reducing/abstaining from alcohol/substance use	☐	☐	☐	☐	☐	☐	☐

Comments

Informational needs

How would you rate your need for support with the following?	1	2	3	4	5	NA	Revisit later
Understanding your:							
Diagnosis	☐	☐	☐	☐	☐	☐	☐
Treatment plan	☐	☐	☐	☐	☐	☐	☐
Medications	☐	☐	☐	☐	☐	☐	☐
Recovery and how this could be enhanced	☐	☐	☐	☐	☐		☐

Comments

Emotional & Psychological needs

How would you rate your need for support with the following?	1	2	3	4	5	NA	Revisit later
Understanding, expressing and managing your emotions (e.g. anger, frustration, overwhelm)	☐	☐	☐	☐	☐		☐
Understanding, expressing and managing your thoughts (e.g. racing thoughts, negative thoughts about self)	☐	☐	☐	☐	☐		☐
Managing psychological symptoms (e.g. anxiety, agitation)	☐	☐	☐	☐	☐		☐
Feeling safe when you experience thoughts of self-harm	☐	☐	☐	☐	☐	☐	☐
Feeling safe when you experience thoughts of suicide	☐	☐	☐	☐	☐	☐	☐
Managing loneliness	☐	☐	☐	☐	☐	☐	☐

Comments

Relationship needs

How would you rate your need for support with the following?	1	2	3	4	5	NA	Revisit later
Discussing your diagnosis and recovery with your:							
Spouse/partner	☐	☐	☐	☐	☐	☐	☐
Family members	☐	☐	☐	☐	☐		☐
Friends	☐	☐	☐	☐	☐		☐
Employer/school authorities	☐	☐	☐	☐	☐	☐	☐
Finding a peer support group (e.g. mental health, online, addiction or physical illness support group)	☐	☐	☐	☐	☐		☐

Comments

Needs
in
Recovery
Assessment

Section 2 - Planning discussion

	Approach for meeting need	Obstacles and barriers to meeting need	Person/organisation available to assist	Action taken (e.g. referral)
**Priority 1:**<br><br>Details:			☐N/A	☐Yes ☐No ☐N/A<br>Details:
**Priority 2:**<br><br>Details:			☐N/A	☐Yes ☐No ☐N/A<br>Details:
**Priority 3:**<br><br>Details:			☐N/A	☐Yes ☐No ☐N/A<br>Details:
**Priority 4:**<br><br>Details:			☐N/A	☐Yes ☐No ☐N/A<br>Details:

Copy of planning discussion provided to service user: Yes ☐ No ☐

Scheduled date of follow-up appointment to discuss needs and progress:____/____/20___ *(This should occur 1-2 weeks after completion of Sections 1 and 2)*

Section 3 - Follow-up discussion

Date of assessment:
Place of assessment:
Service user name:
Service user ID/URN:
Lead mental health clinician:
Others present:

Date:____/____/20___ Place of assessment:

Identified need	What has gone well with meeting this need?	What hasn't gone so well with meeting this need?	What are the next steps for meeting this need?
1.			
2.			
3.			
4.			

Comments:

Copy of follow-up discussion provided to service user: Yes ☐ No ☐
